# Mind over brain, brain over mind: cognitive causes and consequences of controlling brain activity

**DOI:** 10.3389/fnhum.2014.00348

**Published:** 2014-05-27

**Authors:** Elisabeth V. C. Friedrich, Guilherme Wood, Reinhold Scherer, Christa Neuper

**Affiliations:** ^1^Department of Psychology, University of Graz, BioTechMed-GrazGraz, Austria; ^2^Department of Cognitive Science, University of California San DiegoLa Jolla, CA, USA; ^3^Laboratory of Brain-Computer Interfaces, Institute for Knowledge Discovery, Graz University of Technology, BioTechMed-GrazGraz, Austria

**Keywords:** brain-computer interface (BCI), neurofeedback (NF), electroencephalogram (EEG), functional magnetic resonance imaging (fMRI), training protocol, psychological traits, control and its prediction, applications for disabled individuals

Brain activity is considered the physical correlate of mental activity. Accordingly, a change of the state of mind implies a change in the state of the brain and vice versa. This homology between brain activity and cognitive tasks is very difficult to measure due to their very high complexity, temporal and spatial dynamics, as well as individual variability. However, the advancing of technology allows the detection of some states of brain activity with reasonable accuracy and their translation into control messages for devices external to the brain (i.e., brain-computer-interfaces, BCI). Moreover, studies on neurofeedback (NF) show that individuals can learn to modulate their own brain activity based on external feedback and thereby induce changes in cognition and behavior which can be used as therapy for various mental disorders.

Interestingly, roughly about one third of people succeed in controlling computerized devices with brain signals right away; one third gains control after training and one third does not achieve useful control even by using state-of-the-art BCI or NF technology. Various factors have been related to the individual success but to date, no general theoretical framework is available. In this Research Topic, **aspects of the training protocol** such as instructions, task and feedback as well as **psychological traits** such as motivation, mood, locus of control, and empathy are investigated as determinants of BCI or NF performance. Moreover, the brain generates a large amount of coherent spontaneous activity independently of the BCI or NF task at hand which negatively impacts the reliable detection of brain activity patterns. Thus, the mechanisms and networks involved in gaining and maintaining **control over brain activity** as well as **its prediction** are addressed. Finally, as the ultimate goal of our research is to use BCI and NF for communication or control and therapy, respectively, novel **applications for individuals with disabilities or disorders** are discussed.

The first part of the research topic deals with the role of **the training protocol** in BCI and NF. In the hypothesis and theory article of Lotte et al. (2013), problems in current BCI training protocols are identified according to instructional design principles and solutions for improved instruction, task, and feedback are proposed. Kober et al. (2013) report that the failure to describe a specific mental strategy when learning NF is indicative of better performance in an electroencephalographic (EEG)-based NF training paradigm controlled by modulation of the sensorimotor rhythm (SMR). Addressing the impact of the feedback in EEG-based BCIs, Koerner et al. (2014) demonstrate that the presentation of sham positive feedback resulted in different and better classifiable EEG patterns in comparison to sham negative feedback. Thus, the feedback success rate directly influences brain signals.

The findings addressing the impact of **psychological traits** on performance are in line with the above presented results. Subramaniam and Vinogradov (2013) discuss in their review article how positive mood states change brain patterns and improve cognitive performance, which implies that mood states might also influence BCI and NF performance. Moreover, motivation and personality traits might also change brain patterns and performance. In contrast to earlier findings, Kleih and Kübler (2013) do not find a correlation between motivation and performance in an EEG-based P300-BCI. However, these authors report a negative correlation of empathic characteristics and P300 amplitudes. Also, Witte et al. (2013) demonstrate a negative correlation between the locus of control with regard to technology and SMR power in an EEG-based SMR-NF. The authors of both articles conclude that a high degree of empathy as well as high expectations regarding the locus of control might lead to emotional or cognitive overload, which, in turn, leads to lower performance in P300 or SMR-based BCIs. This is in line with the findings of Kober and colleagues addressing spontaneous mental tasks. Thus, a state of positive but not emotionally involved attentive and effortless relaxation might be the optimal state to control both NF and BCI.

Besides external and internal factors influencing performance, neurophysiological as well as peripheral physiological correlates of gaining and maintaining **control of brain activity** are important to address. Ninaus et al. (2013) demonstrate in their study using functional magnetic resonance imaging (fMRI) that the fronto-parietal and cingulo-opercular network which are typically involved in cognitive control is active when participants believe to control a NF. Berman et al. (2013) examine the possibility to control the functionally localized anterior right insular cortex with fMRI-based NF. Also, peripheral physiological signals might have an impact on cognitive control. Pfurtscheller et al. (2013) investigate the interaction between brain and heart and suggest that the changes in heart rate in correlation with motor imagery can be used as indicator of mental effort to improve BCI control.

To **predict the ability to control** a NF or BCI, Halder et al. (2013) performed MRI scans after one session of EEG-based SMR-BCI using motor imagery and report a positive correlation between individual BCI performance and the structural integrity and myelination quality of deep white matter structures. Also using MRI scans, Enriquez-Geppert et al. (2013) show that the volume of the midcingulate cortex as well as volume and concentration of the underlying white matter structures predicts EEG-based NF performance of frontal-midline theta performance. Both studies indicate that there is a neuroanatomical foundation for the aptitude to control a BCI or NF. In contrast to all mentioned research studies so far, Riccio et al. (2013) included individuals with amyotrophic lateral sclerosis to investigate predictor variables of performance in an EEG-based P300-BCI. They conclude that the temporal filtering capacity (i.e., ability to keep the attentional filter active during target selection) is crucial for BCI control.

The last part covers potential **applications** for individuals with disabilities or mental disorders. Risetti et al. (2013) present findings of an EEG-based P300 auditory oddball paradigm to investigate residual unconscious and conscious cognitive function in individuals with a disorder of consciousness. Micoulaud-Franchi et al. (2013) propose in their perspective article to couple repetitive transcranial magnetic stimulation (rTMS) with NF and discuss therapeutic implications and ethical issues.

In summary, this Research Topic illustrates how different factors impact BCI and NF performance and provides new perspectives that need addressing in the future.

## Conflict of interest statement

The authors declare that the research was conducted in the absence of any commercial or financial relationships that could be construed as a potential conflict of interest.
